# The Skeletal Cellular and Molecular Underpinning of the Murine Hindlimb Unloading Model

**DOI:** 10.3389/fphys.2021.749464

**Published:** 2021-10-19

**Authors:** Priyanka Garg, Maura Strigini, Laura Peurière, Laurence Vico, Donata Iandolo

**Affiliations:** INSERM, U1059 Sainbiose, Université Jean Monnet, Mines Saint-Étienne, Université de Lyon, Saint-Étienne, France

**Keywords:** hindlimb unloading, osteocyte, mechanotransduction, bone loss, disuse osteopenia

## Abstract

Bone adaptation to spaceflight results in bone loss at weight bearing sites following the absence of the stimulus represented by ground force. The rodent hindlimb unloading model was designed to mimic the loss of mechanical loading experienced by astronauts in spaceflight to better understand the mechanisms causing this disuse-induced bone loss. The model has also been largely adopted to study disuse osteopenia and therefore to test drugs for its treatment. Loss of trabecular and cortical bone is observed in long bones of hindlimbs in tail-suspended rodents. Over the years, osteocytes have been shown to play a key role in sensing mechanical stress/stimulus *via* the ECM-integrin-cytoskeletal axis and to respond to it by regulating different cytokines such as SOST and RANKL. Colder experimental environments (~20–22°C) below thermoneutral temperatures (~28–32°C) exacerbate bone loss. Hence, it is important to consider the role of environmental temperatures on the experimental outcomes. We provide insights into the cellular and molecular pathways that have been shown to play a role in the hindlimb unloading and recommendations to minimize the effects of conditions that we refer to as confounding factors.

## Introduction

The organism continuously renews its skeleton, by constantly resorbing and building bone tissue throughout its life. If bone resorption outpaces its formation, bone mass and strength are diminished. Bed rest, immobilization, paralysis, and spaceflight all share the common feature of bone loss, detected as reduced bone density at specific bone sites, potentially leading to osteopenia and osteoporosis (Takata and Yasui, 2001). The bone loss seen in such conditions is thought to be primarily caused by loss of mechanical loading (Lau and Guo, 2011). Microgravity causes bone loss especially, though not exclusively, at the weight-bearing sites (Oganov et al., 1992; Vico et al., 2000; Linossier et al., 2017). Similarly, in long term bed rest the loss of ground force reaction is accompanied by reduced muscle contractions and subsequent decrease in bone mineral density at distal femur, distal tibia and patella (Rittweger et al., 2009).

**Figure 1:**
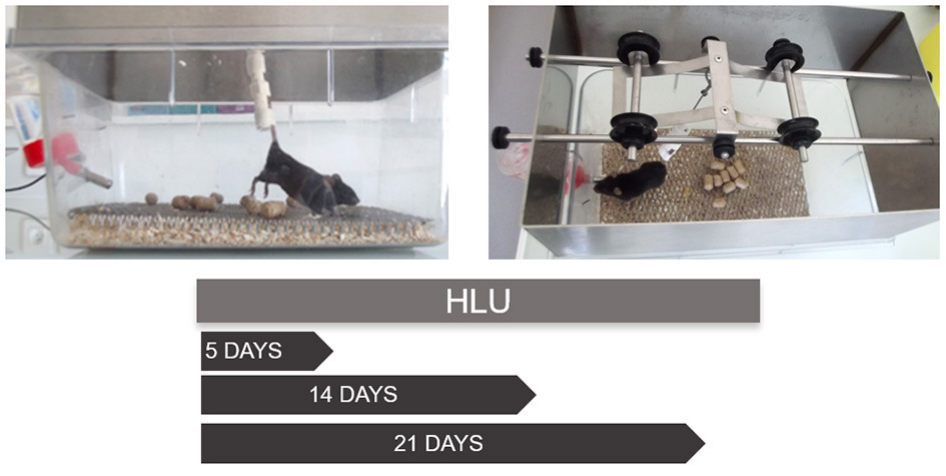
The suspension of the hindlimbs of a C57BL/6J mouse *via* tail suspension and the usual protocol durations. The suspension system consists of the padded rod to which the tail is attached using three tonoplast bandages wrapped at a distance from each other to not cover the tail completely. The rod is then hooked to a freely rotating swivel fixed to a pulley sliding on a roller axis to allow the mouse to freely rotate in 360° axis. The mouse is singly caged and allowed to move on its forelimbs with the support of the grid below. The angle of unloading is maintained at 30° from the ground. The periods of suspension may vary based on the experimental requirements. The different periods of suspension allow one to follow the kinetics of bone adaptation to unloading. At all-time points adaptation can be identified at different levels: molecular (gene expression profiling), cellular (histomorphometry) and tissular (X-ray tomography). Although tail traction with orthopedic tape is the preferred method of suspension, other methods including use of a body harness to stimulate partial weight bearing have been illustrated (Wagner et al., 2010).

If bed rest volunteers have been used to understand disuse osteopenia in clinical studies, the need to better understand the organismal response to microgravity resulted in the development of the preclinical rodent hindlimb unloading (HLU) model in the 1970s (Morey, 1979) (Figure 1). The HLU model as such has been extensively detailed by Morey-Holton and Globus (Morey-Holton and Globus, 1998, 2002; Globus and Morey-Holton, 2016). Briefly, the model encompasses the unloading of the hindquarters of the rodent *via* tail suspension while the animal is left free to walk on its forelimbs. This position mimics the cephalic fluid shift and the decrease in mechanical loading on lower limbs experienced by astronauts during spaceflight (Morey-Holton and Globus, 1998), with the human equivalent being head-down tilt bed rest.

The animal analog has now been adopted as a model to study muscle atrophy and disuse osteoporosis on ground conditions. Several alternatives to it exist such as tenotomy, neurectomy, botulin-induced paralysis, and unilateral limb casting (Komori, 2015). HLU has several benefits such as requiring minimal specialized equipment and not requiring a surgical intervention, unlike tenotomy or neurectomy. When compared to unilateral limb casting, HLU also additionally diminishes the mechanical load (Speacht et al., 2018). Unlike neurectomy, limb unloading itself is partially reversible with the recovery of bone mass being achieved to a certain extent when rodents are let free to use the four limbs again. HLU can be applied to rodents of the age, sex or genetic make-up of interest, allowing the modeling of the various complex clinical scenarios of bone loss observed in humans and the testing of anti-osteopenic drug therapies.

We want to bring to attention of the reader that although the HLU was developed in rats (reviewed in Morey-Holton et al., 2005) it has been well-adapted to mice. We have chosen primarily to focus on mice-based experiments rather than rats as mice offer certain advantages. Beyond ease of handling, their smaller weight allowing for smaller cages and for longer suspension periods. Also, increased availability of transgenic and mutant strains of mice makes it possible the study of specific molecular mechanisms (Ishijima et al., 2001; Iwaniec et al., 2005; Maurel et al., 2016; Yang et al., 2020). Moreover, rats achieve skeletal maturity toward the end of their life (Roach et al., 2003). Therefore, adult mice are preferred to adult but continuously growing rats for suspension models to study skeletal systems, in growing rats the loss of bone is attributed to a failure in increased bone formation and growth, where as in adult mice there is increased bone resorption and net bone loss, which is similar and more relevant to the case of humans in spaceflight (Globus and Morey-Holton, 2016).

This review sets to explore the cellular and molecular underpinnings of the response of bone resident cells to mechanical unloading in HLU. We examine the involved pathways and the role played by the different bone cells (with a particular focus on osteocytes). In addition, we highlight the effects of several key parameters of the experimental setup (e.g., environmental conditions) on the observed outcomes.

## Bone Tissue Changes in HLU

HLU is reported to lead to a number of changes in bone structure both at the cortical and the trabecular levels. The majority of the studies adopting HLU have highlighted a more prominent loss in bone mass and architecture in the trabecular compartment secondary to increased osteoclastic resorption and decreased bone formation. Bone loss is reflected by a decrease in the relative trabecular bone volume (Bone Volume/Total volume) and it is connected to a disequilibrium in bone remodeling. This unbalance is apparent when measuring key static and dynamic bone parameters by histomorphometry, such as bone surface covered by osteoclasts (osteoclast surface over Bone surface, Oc.S/BS), osteoclast number per Bone surface (Oc.N/BS), mineralized trabecular surface per bone surface (MS/BS), mineral apposition rate (MAR), and bone formation rate (BFR) (Komori, 2015). HLU results in increased Oc.S/BS and Oc.N/BS and reduced MS/BS, MAR and BFR. Skeletal unloading also induces cortical thinning (Iwaniec et al., 2005; Morey-Holton et al., 2005; Speacht et al., 2018). Altered bone deposition and resorption patterns contribute to these effects with changes in both periosteum and endosteum formation rates that are not uniform along the bone length (Yang et al., 2020).

These results are consistent with those from *ex vivo* cultures of bone cells derived from tail suspended rats showing reduced osteoblasts' osteogenic potential with a decrease in the number of alkaline phosphatase (ALP)-positive colonies. Notably, an increase in tartrate resistant acid phosphatase (TRAP) positive cells was reported as well, therefore hinting at an increased osteoclastic activity (Maurel et al., 2016). Table 1 summarizes the bone trabecular and cortical changes seen in the hindlimbs of tail suspended mice.

**Table 1:** Summary of trabecular and cortical changes in mice in HLU.

**Experimental condition**	**Trabecular differences**	**Cortical differences**	**References**
17-week old C57BL6 mice 14 days HLU 22°C 1-week acclimatization	Femoral BV/TV: 15%↓ Femur Tb.Th.: 13% ↓	Not measured	Amblard et al., 2003
11-week old C57BL6 mice 21 days HLU 22°C No acclimatization	Tibial BV/TV: 28.2% ↓ Tibial Tb.Th: 11% ↓	Tibial Ct.Th: 21% ↓ Tibial Ct.Ar: 22% ↓	Ellman et al., 2014
12-week old C57BL6 mice 21 days HLU 22°C No acclimatization	Tibial trabecular BMD: ~18% ↓	No differences reported in cortical BMD	Kawao et al., 2018
16-week-old WBB6F mice 14 days HLU 32°C 12 weeks acclimatization	Femoral BV/TV: ~20% ↓, Femoral Tb.Th: ~9% ↓	Not measured	Keune et al., 2017
17-week old C57BL6 mice 14 days HLU 22°C No acclimatization	Femoral BV/TV: >60% ↓ Femoral Tb.Th: ~30% ↓	Femoral Ct.Th: ~23% ↓ Femoral Ct.Ar: ~32% ↓	Lin et al., 2009
7-week old BALB/c mice 28 days HLU 22°C 1-week acclimatization	Femoral BV/TV: ~75% ↓	Femoral bone volume: ~13%	Saxena et al., 2011
6-month C57BL6 mice 7, 14 and 28 days HLU 22°C Acclimatization not stated	Femoral BV/TV: ~28% ↓ Femoral Tb.Th: ~11% ↓	Not measured	Shahnazari et al., 2012
52-day old mice 14 days HLU 22°C Acclimatization not stated	Trabecular parameters not measured	Femoral Ct.Th: 25% ↓ Femoral Ct.Ar: 16% ↓	Simske et al., 1992
12-week old C57BL6 mice 21 days HLU 22°C Acclimatization not stated	Femoral BV/TV: ~29% ↓ Femoral Tb.Th: ~12% ↓	Femoral Ct.Th: ~19% ↓ Femoral Ct.Ar: ~17% ↓	Spatz et al., 2013
8-week old C57BL6 mice 28 days HLU Acclimatization not stated	Femoral BV/TV: ~15% ↓ Femoral trabecular BMD: ~22% ↓	Femoral Ct.Th: ~10% ↓	Colaianni et al., 2017
14-week old C57BL6 mice 14 days HLU 22°C Acclimatization 2 weeks	Tibial Tb.Th.: 11% ↓	Tibial Ct.Th: ~17% ↓	Steczina et al., 2020
8-week old ddY mice 21 days HLU 23°C 1-week Acclimatization	Femoral BV/TV: ~13% ↓ Femoral trabecular BMD: ~20% ↓	No differences reported in cortical BMD	Tousen et al., 2020

*Details on the experimental procedures are reported in reference to the mouse strain, the temperature, and the acclimatization phase. The arrows pointing downwards correspond to a reduction in the described parameter. BV/TV, Bone Volume/Total Volume; BMD, Bone mineral density; Tb.Th, Trabecular Thickness; Ct.Th, Cortical Thickness; Ct.Ar, Cortical Area*.

## Osteocyte Lacuno-Canalicular Space and Mechanosensors

Frost proposed that bone has an intrinsic regulatory mechanism, that he called the mechanostat. This regulatory “machinery” would respond to mechanical stimulation by adapting bone morphological, biochemical, and physical properties to serve its mechanical function in the most economical way. This model would anticipate an increased net bone resorption below a threshold of mechanical stimulation/use and net bone formation above a specific threshold (Frost, 1987). Frost's mechanostat theory provided a conceptual framework to rationalize bone adaptation to mechanical stimuli but it did not clarify which cells would sense the loading or how this information would be transmitted to bone forming and bone resorbing cells.

Eventually, osteoblasts, osteoclasts, and osteocytes, the three key cell types in bone, and their precursors have all been demonstrated to contribute, directly or indirectly, to bone homeostasis. In particular, osteocytes stand out as the key orchestrators of bone remodeling *via* regulation of osteoblastic bone mineralized matrix deposition and osteoclastic bone resorption (Bonewald, 2006, 2011).

**Figure 2:**
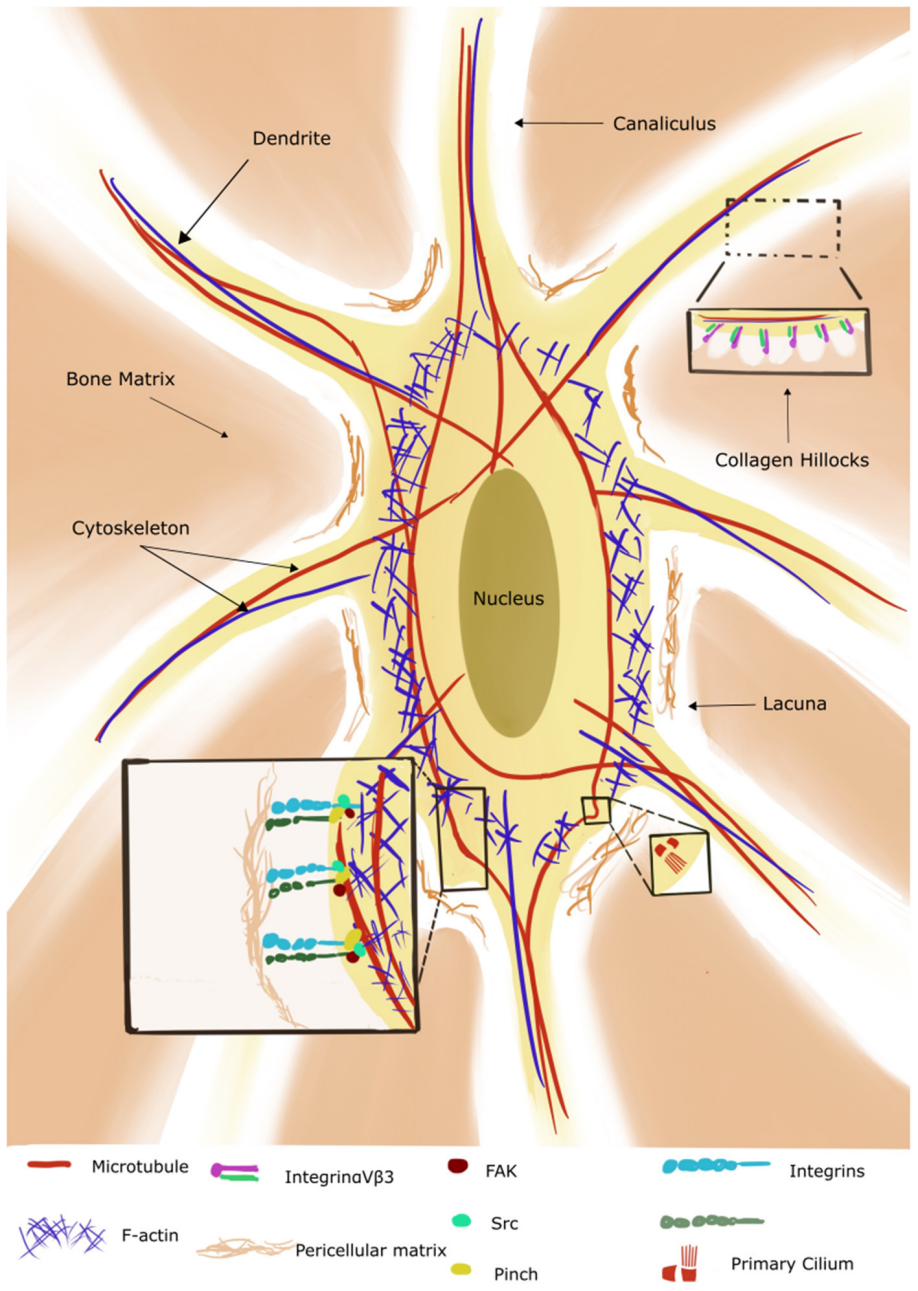
Illustration of an osteocyte with its lacuno-canalicular system (adapted from Qin et al., 2020). The cytoskeleton consists of microtubules (red) which extend to the primary cilium, actin (blue) and intermediate filaments (not shown in the figure). The bone osteoid forms collagen hillocks at the canaliculi. Integrins are present on the cell body and dendrites/osteocyte processes and interact with the pericellular matrix and cytoskeleton *via* the focal adhesion components (only three shown for simplicity). (Reproduced with permission: http://creativecommons.org/licenses/by/4.0/).

Osteocytes reside in cavities called lacunae within the mineralized matrix, and send extensions called dendrites or osteocytic processes through the canaliculi (tunnels) crossing the bone matrix. The lacunae together with the canaliculi form the lacuno-canalicular space (LCS), that is filled with a fluid responsible for transporting solutes to and from the osteocyte (Figure 2). Dye injection into the tail vein of a mouse shows passage of the dye from circulation into the LCS within minutes (Knothe Tate et al., 2000; Price et al., 2011). Extravascular pressure drives the baseline flow of the canalicular fluid, but rapid alterations can occur in the flow due to changes in the mechanical loading [theoretically modeled by (Weinbaum et al., 1994)]. Later revisions of such model are detailed in a recent review by Hadida and Marchat (2020).

In this section we focus on the mechanosensors that have been explored in conjunction with HLU. The complex architecture of this space allows the transmission of the mechanical information from the scale of the fluid movement to the 3D matrix of the osteocyte cytoskeleton *via* transmembrane molecules like the protein Piezo1 (as reviewed in Qin et al., 2020). Piezo is a family of mechanosensitive cell membrane ion channels expressed in osteoblasts and osteocytes. It has been demonstrated that Piezo1 opens in response to mechanical stimuli and allows the entry of calcium ions into the cytoplasm (Coste et al., 2010). Mice with osteoblasts and osteocytes deficient in the Piezo channel show decreased trabecular bone volume, cortical thickness, and increased osteoclasts. However, these mice do not undergo bone loss when used in HLU experiments, highlighting the role of Piezo-1 as a key regulator in the response to mechanical loading (Sun et al., 2019; Wang et al., 2020). The ECM-integrin-cytoskeleton axis is crucial to mechanotransduction (reviewed in Yavropoulou and Yovos, 2016). The pericellular matrix of osteocytes forms hillock structures called “collagen hillocks” connecting the dendrites to the matrix (Figure 2). The matrix is in turn connected with the integrin-focal adhesion complexes in the cell body. Mechanical strain induces assembly of the focal adhesion molecules in association with integrins, and activation of the focal adhesion kinase (FAK) and the Src pathways ultimately resulting in the activation of the phosphoinositide 3-kinase (PI3K) and the mitogen activated protein kinase (MAPK) pathways (Marie et al., 2014). The MAPK pathways then specifically upregulate the expression levels of runt-related transcription factor 2 (RUNX2), Osterix, and activating transcription factor 4 (ATF4) promoting osteoblastogenesis (Franceschi and Ge, 2017). The three major cytoskeletal components in the osteocyte include actin filaments, which extend into the dendrites and are essential for osteocyte integrity, intermediate filaments like vimentin, whose levels are sensitive to mechanical stimulation, and microtubules, with the latter extending into the primary cilium (Figure 2) (Tanaka-Kamioka et al., 1998; Klein-Nulend et al., 2012). Primary cilia are “solitary” organelles projecting from the cell surface and mainly functioning as chemo- and mechanosensors. Kwon et al. explored their way of functioning in osteocytes and demonstrated that primary cilia bend under physiological levels of flow with a consequent decrease in cAMP levels mediated by adenylyl cyclase 6. They speculated that this decrease is transient and followed by the accumulation of cAMP which subsequently causes transcriptional changes in cyclooxygenase 2 (COX-2) and thus prostaglandin 2 (PGE2) expression (Kwon et al., 2010). PGE2 is a rapidly induced signaling molecule which is released in response to fluid flow shear stress and further acts *via* PKA, β-catenin pathways (Kamel et al., 2010; Kitase et al., 2010). However, it should be mentioned here that only a small percentage of bone cells (4%) have been found to carry primary cilia *in vivo*, indicating that the primary cilia probably function with other mechanosensory systems/organelles (Coughlin et al., 2015). The osteocytes communicate with each other and with the surrounding cells *via* gap junctions containing the protein connexin 43 (Cx43). Gap junctions enable quicker propagation of secondary messengers to adjacent cells, creating a functional syncytium throughout the bone (Buo and Stains, 2014). Gap junctions open in response to mechanical stress with resultant release of PGE2, and activation of the PI3K/AKT and cyclic adenosine monophosphate/protein kinase A pathways (cAMP/PKA) (Cherian et al., 2003; Xia et al., 2010). Cx43 knockout mice show an osteopenic phenotype with decrease in cortical BMD, thickness and increased porosity (Lloyd et al., 2012). Interestingly, the selective deletion of Cx43 in osteocytes also desensitized bone to 3 weeks of hindlimb unloading. In these animals, no increase in osteoclastic resorption was seen resulting in no decline in trabecular bone volume, thickness and density, indicating the inability of Cx43^−/−^ osteocytes to efficiently detect, “communicate” or respond to unloading. Thus, deletion of Cx43 affects not only the physiological bone phenotype but also its response to loading (Lloyd et al., 2013).

Therefore, it can be derived that mechanical stimuli exert their stimulatory role by primarily modifying fluid flow which can induce matrix deformation, trigger membrane mechanoreceptors and induce cytoskeletal responses (Kitase et al., 2010).

Although the fluid flow theory provides a mechanical framework and allows a mechanistic analysis in terms of cell responses, it is not the only validated/critical mechanism for bone adaptation to mechanical loading and is not limited to the mechanosensors mentioned above. Also, comprehensive explanation of the biomolecular responses to perturbations in the fluid microenvironment in the HLU model remain elusive. To better understand the role played by mechanotransduction in bone health, it is important to further review the molecular pathways it relies on.

## YAP/TAZ Pathway

YAP (Yes associated protein) and TAZ (Transcriptional factor with PDZ binding motif) are transcriptional factors that are increasingly being investigated for their role in cellular processes and have been found implicated in a number of physio-pathological conditions (e.g., arthritis, arthrosis, tumor metastases). Their activity can also be regulated by the rigidity and deformation of the ECM as it has been demonstrated in various cell types including the osteoblastic lineage (reviewed in Panciera et al., 2017). Their function is dependent on the integrity of actin cytoskeleton and requires Ras homologous protein (Rho) activity (Dupont et al., 2011). *In vitro* studies show ECM rigidity causes activation of the integrin-Rho pathways in cells which undergo cytoskeletal reorganization (e.g., increased polymerization, contractility, and stress fiber pooling) and consequent nuclear translocation of the YAP-TAZ complex, promoting differentiation of human mesenchymal stem cells (Kegelman et al., 2020).

Regarding their role in the regulation of osteoblastogenesis, data on the effects of YAP/TAZ signaling is contradictory and it appears that they have opposing effects at different stages of osteoblast differentiation. The concomitant deletion of YAP and TAZ in osteoprogenitor cells (Prx-1 Cre targeted mice lacking two copies of TAZ and one copy of YAP) resulted in increased osteoblast differentiation, whereas their deletion in mature osteoblasts/osteocytes in Dmp1-Cre mice reduced bone formation and also increased osteoclast number (Xiong et al., 2018). *In vivo* studies show that Piezo1 regulates YAP pathways in bone cells: mechanical stimulation transduced by Piezo1 leads to YAP nuclear accumulation and downstream of Ca^2+^ influx and Calcineurin activation, concomitant to Wnt/beta-Catenin pathway (Zhou et al., 2020). In Piezo1 conditional knockout mice, reduced nuclear localization of YAP with resultant osteoporosis. The same knockout mice, when suspended, resist bone loss (Wang et al., 2020). Atrophied muscle fibers from suspension do show a decrease in YAP protein which increases on reloading (Brooks et al., 2018). As such it would be interesting to look at the changes in YAP/TAZ pathways in bone during mechanical unloading. It has been shown that the pathway also interacts with Wnt canonical pathways (see below) (Azzolin et al., 2014).

*In vitro* studies show that YAP can promote or inhibit osteogenic differentiation in bone marrow mesenchymal stem cells (BMSCs) (Sen et al., 2015; Liu et al., 2019), whereas TAZ was shown to promote osteogenic differentiation in BMSC and MC3T3 cultures (Kim et al., 2014; Feng et al., 2015).

Overall, the YAP/TAZ pathway seems to be an important molecular pathway for the translation of mechanical stimuli into biochemical signals. However, further research in both *in vitro* and *in vivo* models is required to understand its dynamic role in response to unloading.

## Wnt Pathway

Wingless-related integration site (Wnt) pathways are evolutionary conserved pathways comprising of a family of 19 glycoproteins which regulate several crucial aspects of cell fate and migration as well as organogenesis (reviewed in Komiya and Habas, 2008).

Wnt pathways have been known to positively contribute to bone mass *via* a number of mechanisms which include stem cell renewal, induction of osteoblastogenesis and prevention of both osteoblast and osteocyte death (Krishnan et al., 2006; Moorer and Riddle, 2018).

Briefly, in Wnt pathway OFF state, a destruction complex consisting of APC (Adenomatosis Polyposis Coli), axin, GSK3 (glycogen synthase kinase 3), and casein kinase 1 (CK1) is formed and it phosphorylates β-catenin therefore leading to its degradation in the proteasome. In its ON state, Wnt ligand binds to the frizzled receptors, LRP5/6 (low-density-lipoprotein-related protein 5/6), causing the inactivation of GSK3 *via* Disheveled (Dsh), a key component of Wnt-signaling pathways (Lerner and Ohlsson, 2015). This results in the accumulation and nuclear translocation of β-catenin. Here, β-catenin displaces the transcriptional co-repressors bound to TCF/LEF (t-cell factor/lymphoid enhanced factor) and recruits co-activators, regulating the expression of target genes such as cyclinD, c-Myc, peroxisome proliferator activated receptor (PPAR) and axin2, which participates in a negative feedback loop and limits the duration of Wnt signaling pathway (Jho et al., 2002; Krishnan et al., 2006).

Increased expression of Wnt target genes (i.e., Wnt10B, SFRP1, cyclin D1) was reported in response to mechanical loading both *in vivo* and *in vitro* (Robinson et al., 2006). On the contrary, decreased mRNA expression of LRP6 and β-catenin was seen in rats after 4 weeks of tail-suspension (Jia et al., 2019). Mice with an activating point mutation in the Wnt coreceptor LRP5 have high bone mass and they were found to be resistant to bone loss induced by hindlimb unloading (Niziolek et al., 2015). Also, artificial stabilization of β-catenin in osteocytes prevented the disuse-induced bone loss in unloaded mice (Bullock et al., 2019). These results imply an important role for Wnt/β-catenin in bone response to mechanical stimulation.

## Secretory Signaling Proteins

**Sclerostin** is a protein encoded by the SOST gene and secreted mainly by mature osteocytes. SOST knockout mice exhibit a high bone mass phenotype with increased bone mineral density and bone strength due to increased bone formation. In humans, mutations in this gene are associated with rare genetic disorders associated with high bone mass, as sclerosteosis and van Buchem disease (Balemans et al., 2001; Li et al., 2008). Sclerostin is an antagonist of the Wnt downstream signaling pathway and it acts by binding to the LRP5/6 receptors (Li et al., 2005). Thus, it is a negative regulator of bone formation and it was shown to respond to mechanical loading. *In vivo* mRNA levels of sclerostin are reduced in the ulnar cortex of mice exposed to loading, whereas they are increased in the tibia of tail suspended mice at day 3 of suspension but subside to non-significant levels at day 7 (Robling et al., 2008). However, some experiments show a more complex pattern of SOST expression, with its levels varying with anatomical site. Unloaded hindlimbs (tibiae) of 3 month old rats showed a decrease in SOST expression in metaphyseal cortical bone, and upregulated SOST levels in diaphyseal bone (Macias et al., 2013).

*In vitro* experiments demonstrated that osteocyte cell lines, Ocy454 cells, when subjected to simulated microgravity, as achieved in the NASA rotating wall bioreactors, showed a significant increase in SOST expression when compared to the static controls (Spatz et al., 2015). Inversely, Ocy454 responded to fluid shear stress (FSS) (Lyons et al., 2017) by reducing SOST levels, de-repressing the Wnt signaling pathways. This occurs by a rapid lysosomal degradation of sclerostin within 5 min of exposure to an anabolic stimulus like FSS (Gould et al., 2021).

**DKK1** is another antagonist of the Wnt pathway, acting by binding directly to LRP5/6. Its expression levels in bone were found to be reduced upon ulnar loading (Bafico et al., 2001), but no significant changes were seen on hindlimb unloading (Robling et al., 2008).

Both SOST and DKK1 antagonism using monoclonal antibodies has emerged as a therapeutic approach to treat osteoporosis (Ke et al., 2012). Romosozumab, a humanized monoclonal antibody against sclerostin, has been shown to reduce the risk of vertebral fractures in postmenopausal women and is already in use in United States and European Union (Paik and Scott, 2020).

**RANK-RANKL** signaling is necessary for the differentiation and activation of osteoclasts and subsequent bone resorption (reviewed in Ono et al., 2020). RANKL was believed to be secreted primarily by osteoblasts but the deletion of RANKL in late osteoblasts/osteocytes (Tnfsf11-floxed mice crossed with Dmp 1-Cre) showed that RANKL was mostly produced at this stage in adult mice (Xiong et al., 2015). In the cancellous bones of distal femurs of hindlimb unloaded rats elevated levels of pro-inflammatory cytokines (e.g., IL-1, TNF-α) were reported to lead to an increase in RANKL (Metzger et al., 2017). Several authors have described an increase in RANKL production by osteocytes neighboring osteocytes that were believed to undergo apoptosis as a response to loss of mechanical load or conversely excessive loading with resultant microfractures (reviewed in Xiong and O'Brien, 2012). Increase in bone resorption following osteocyte apoptosis has also been reported in cortical bone of OVX mice (Emerton et al., 2010).

Femurs of suspended mice showed an increase in osteocyte apoptosis with increase in RANKL and bone resorption (Cabahug-Zuckerman et al., 2016). However, Plotkin et al. (2015) demonstrated that bone resorption and bone loss occur even after blocking of osteocyte apoptosis using the inhibitor of apoptosis IG9402 (bisphosphate analog that maintains osteoblast and osteocyte viability). Other studies have also demonstrated that spaceflight and HLU induced bone loss occurs even in the absence of osteocyte apoptosis (Blaber et al., 2013; Farley et al., 2020). Moreover, the results from papers reporting apoptotic osteocytes should be treated with caution because they derive from immunohistochemical analyses run on thin (5 μm) sections, potentially leading to false positives when counting empty osteocyte *lacunae* (Jilka et al., 2013). False positives have also been reported for activated caspase 3 immunostaining and TUNEL staining with certain decalcification and pre-labeling techniques (Emans et al., 2005).

Also, counting empty *lacunae* does not give any information on the process causing cell death. It would therefore not be possible to distinguish between apoptosis or senescence. Further research is therefore warranted.

## Bone Mediators of Energy Metabolism

Altered glucose metabolism, including glucose intolerance and insulin resistance, has been documented in astronauts in spaceflight (Stein et al., 1994; Hughson et al., 2016). Similarly, altered glucose metabolism was also seen in ground-based analogs such as head-down bed rest and dry immersion (Heer et al., 2014; De Abreu et al., 2017; Linossier et al., 2017). In hindlimb unloaded mice, fasting glucose levels are higher compared to control mice and insulin resistance is seen after 3 weeks from the beginning of the unloading (Wang et al., 2019). The cause for this metabolic dysfunction is not well-understood but maybe linked to altered levels/functions of osteokines and myokines in HLU.

The osteokine **osteocalcin** (Ocn) is expressed by mature osteoblasts and considered a marker of bone formation. Its expression is decreased during tail suspension and upregulated during mechanical loading (Han et al., 2018). Osteocalcin has been shown to promote the uptake of glucose in muscles at the onset of exercise and enhance the oxidation of glucose and fatty acids to be used by the muscle fibers (Mera et al., 2016). An Ocn^−/−^ mouse line generated by the Karsenty's group shows impaired glucose metabolism, hinting at a role for osteocalcin in this metabolism (Lee et al., 2007). However, these results were questioned by two recent, independent studies using different Ocn^−/−^ mouse lines (Diegel et al., 2020; Komori, 2020). The studies cast doubt on the metabolic roles of osteocalcin as the OCN^−/−^ mice generated demonstrate that OCN is involved in bone quality and collagen maturity but has no effect on glucose metabolism or body weight (Diegel et al., 2020).

**Lipocalin 2** (Lcn2) is expressed in adipose tissue, earning it the title of an adipokine, but it is also expressed by osteoblasts and plays a role in energy metabolism. The serum levels of Lcn2 in healthy volunteers in a prolonged bed rest study (15 days) were found to be elevated (Rucci et al., 2015). In mice, inactivation of lipocalin 2 in osteoblasts results in glucose intolerance and insulin resistance following an increase in food intake (Mosialou et al., 2017). Rucci et al. (2015) demonstrated an increase in lipocalin mRNA expression in bones (distal femur) of mice that had been suspended by their tail for 3 weeks, hypothesizing its role as a novel mechanoresponsive/mechanosensor gene. Transgenic mice overexpressing LCN2 in bone show a decrease in bone mass due to a negative effect on growth plate, decreased osteoblast differentiation and increased osteoclastic resorption (Costa et al., 2013). However, global deletion of lipocalin 2 demonstrated an osteopenic phenotype in mice, with lower trabecular bone volume (Capulli et al., 2018).

The myokine **irisin** is the cleavage product of the fibronectin type III domain-containing protein 5 (FNDC5) being secreted by muscles post exercise and it was identified for its role in the browning of white adipose tissues (Boström et al., 2012). Irisin was found to be significantly decreased in the soleus muscle in HLU mice and this decrease had a positive correlation to trabecular bone mineral density (BMD) (Kawao et al., 2018). Treatment of tail-suspended mice with r-irisin ameliorates disuse-induced osteoporosis, shown to be due to decrease in osteocyte apoptosis (Colaianni et al., 2017; Colucci et al., 2020). Irisin upregulated Opg (osteoprotegerin) in an *in vitro* 3D co-culture system of osteoblasts, osteoclasts and endothelial cells and prevented the downregulation of osteoblastic key transcription factors induced by microgravity (Colucci et al., 2020). However, Estell et al. (2020) showed that irisin can also directly act on osteoclasts to increase bone resorption. These results were obtained with lower doses and continuous administration of irisin when compared to experiments from Grano's lab (Colaianni et al., 2015), and they further support its role in bone remodeling probably as a counter regulatory hormone like PTH (parathyroid hormone). Thus, dosing and timing of irisin can be important determinants of its physiological impact on skeletal tissues.

It should be remembered that the HLU model is inherently stressful for mice and altered corticosteroids can mediate the metabolic changes (Pasieka and Rafacho, 2016). There have been contradictory findings on measures of stress in the HLU model in different laboratories (Morey-Holton and Globus, 2002). A few studies show an initial peak in cortisol levels which later reached basal levels (Steffen and Musacchia, 1987; Sugiyama et al., 2006). However, there are also studies reporting no changes in cortisol levels after unloading (Gaignier et al., 2014). The circadian rhythm of the glucocorticoid peak levels in blood may be a cause for this variability (Yang et al., 2017).

## Confounding Factors And Recommendations

Although no model is universal, it is important to consider the effects of so-called confounding factors while setting the experimental design to guarantee reproducibility and repeatability.

### Housing Temperature

Ambient temperature can affect net energy balance and lead to unpredictable outcomes in metabolic data, in isolated animals especially. An inverse relationship between food intake and housing temperatures has been reported (DeRuisseau et al., 2004). In mice, widely used standard vivarium temperature (20–22°C) conditions cause an increase in energy expenditure with a shift toward increased glucose utilization by Brown adipose Tissue (BAT) for non-shivering thermogenesis (David et al., 2013). Ideally, for mammals, the housing/environmental temperatures should be such that the energy expenditure is 1.6–1.7-fold the basal metabolic rate (Speakman and Keijer, 2012). Fischer et al. (2017) believe that the energy expenditure in mice at 21°C is almost three times higher than the basal metabolic rate and that, therefore, at this temperature they are under considerable metabolic stress (Fischer et al., 2017). C57BL/6J mice show a preference for higher temperatures especially during maintenance and inactive behaviors (Gaskill et al., 2009). Social housing at room temperature results in huddling of mice to conserve heat, or nesting in cases of social isolation, but these behaviors do not completely alleviate cold stress (Maher et al., 2015). Thus, housing temperatures are a critical parameter to consider while conducting these experiments. Cold stress is a major confounding factor and needs to be addressed and taken into consideration by the scientific community when drawing conclusive remarks from experiments carried out in these conditions. As a consequence, an ideal temperature to allow mice to alleviate cold-induced thermogenesis would be 28–29°C (Škop et al., 2020).

Studies show that housing mice individually at 22°C results in premature cancellous bone loss which is not seen in thermoneutral conditions (28–32°C) (Patel et al., 2012; Iwaniec et al., 2016; Martin et al., 2019). HLU experiments that were conducted at 28–32°C showed a decrease in cancellous bone volume in distal femur in suspended mice (Keune et al., 2017, 2019; Farley et al., 2020). Though they did not conduct the same experiments at standard temperature in parallel, their results show trabecular bone loss which is lesser than in comparable experiments at 22°C (Amblard et al., 2003; Lin et al., 2009).

Environmental temperature can affect bone remodeling, and exposure of growing mice to higher temperatures has shown to lengthen long bones (Racine et al., 2018). In a recent paper by Chevalier et al., warmth exposure (34°C) was reported to protect against ovariectomy-induced bone loss in mice. However, the same authors reported that the protective effect of warmth exposure was abolished when microbiota was depleted. Similarly, transplantation of warm-adapted microbiota (i.e., from male mice exposed to warm temperatures for 4 weeks into young male mice kept at room temperature) led to a higher cortical bone volume in the experimental mice (Chevalier et al., 2020). The increasing volume of research on the microbial-skeletal axis opens up new perspectives and likely possibility of new treatments (Behera et al., 2020).

### Recommendations

**Figure 3:**
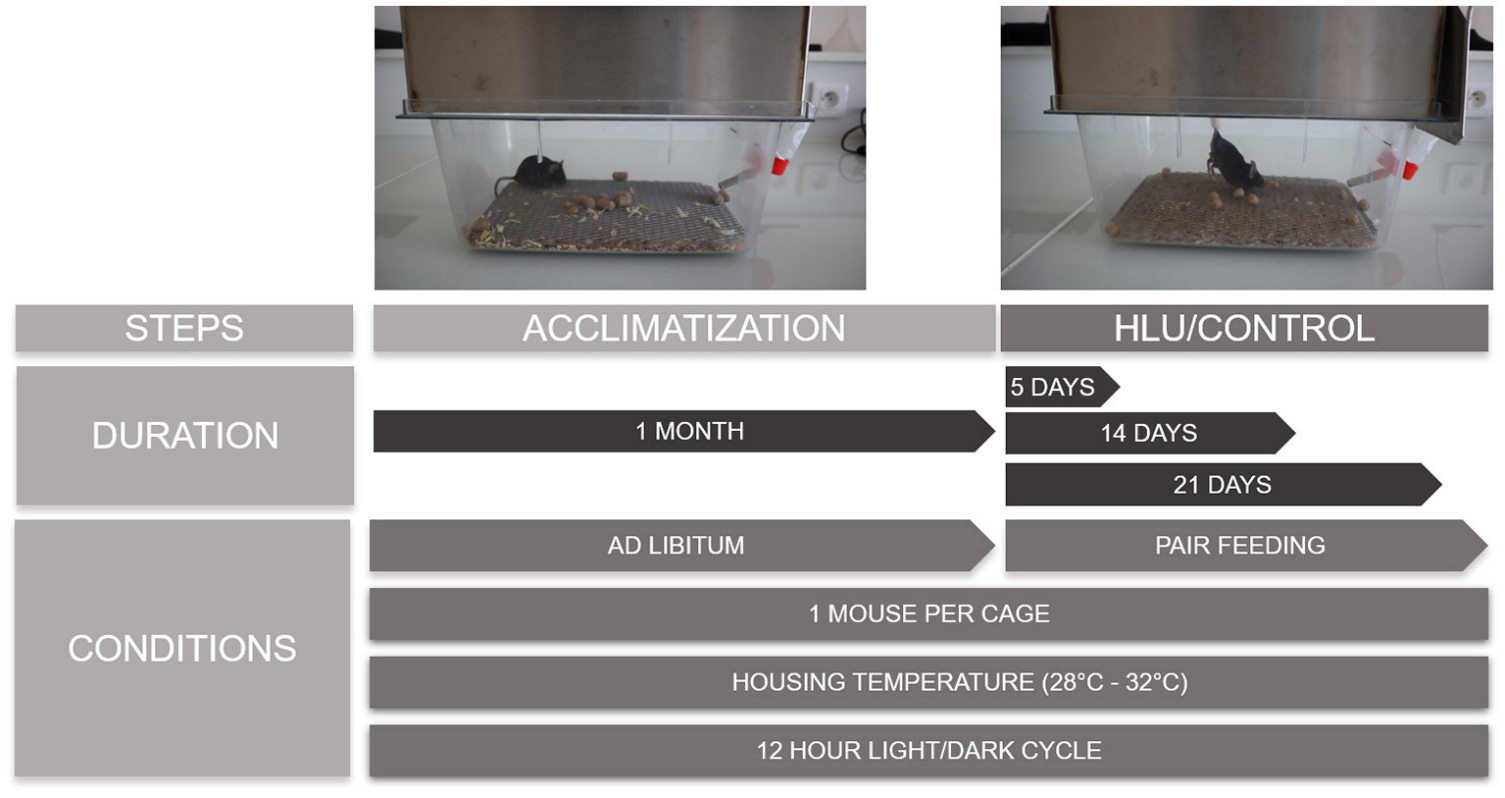
The scheme is reporting pictures of a C57BL/6J mouse during the acclimatization and suspension periods and the conditions and duration of each phase. Sufficiently long periods of acclimatization with *ad libitum* food and water have to be guaranteed, followed by the required periods of suspension where the control mice are pair fed with the suspended ones. Mice are singly housed without straw at thermoneutral temperatures of 28–32°C with 12-h light and dark cycles.

Following are recommendations to consider when setting up an HLU experiment using mice. Experimental details are summarized in Figure 3.

### Mice Genotype

C57BL/6 (B6) is the most commonly used strain as it demonstrates a rapid bone loss to tail suspension (Amblard et al., 2003). It exhibits greater bone mehanosensitiviy and is widely used in bone loss studies (Kodama et al., 2000). B6 mice have lower BMD, lower osteoblastic acitivity but higher osteoclastic resorption when compared to C3H/HeJ mice (Linkhart et al., 1999).

### Age

Mice reach skeletal maturity before 6 months of age and show a pattern of bone loss with aging similar to that in humans (Somerville et al., 2004). Mice total bone mass peaks when they are 4 months old, when it is considered to be representative of the skeletal maturity in human young adults (Beamer et al., 1996). Therefore, depending on the research question an appropriate age of the mice needs to be used. Since the growing skeleton in younger mice can have confounding effects on the results from tail suspension (Simske et al., 1990), a basal control group should be needed and sacrificed at the same time points of the experimental groups.

### Feeding Conditions

During the acclimitization period, the mice are fed *ad libitum*. The control mice tend to eat more than the suspended mice when provided with food *ad libitum*, at least at the beginning of the suspension period. Since dietary restrictions can influence BMD and cause an expansion of the BMAT (Devlin, 2011; Cawthorn et al., 2016), it is necessary to eliminate a possible difference in food intake between the suspended and control groups. This is achieved by pair feeding the control mice to weight matched suspension group.

### Control Conditions

Since we want identical conditions to the experimental group, the control mice are also housed singly without any straw or wood. This is not without consequences and can lead to stress in mice as they cannot huddle to warm up (Tahimic et al., 2019). A grid is placed over the litter to allow easy mobility for the suspended mice and the same should be done for controls as well. Without the grid, the control mice tend to nest in the litter which could create a potential difference of environment between the control and suspended mice. Control mice when attached to the suspension system without unloading show a tendency to chew off the tonoplast bandage/tail. It is therefore suggested to avoid the suspension system in the control groups. Each control mouse are fed the same amount of food as consumed by the weight paired suspended mouse on the previous day.

### Acclimatization and Experimental Duration

Periods of suspension may vary based on whether early or later events are being looked at, but usually they vary between 5, 14, and 21 days and in some studies 28 days. It is important to standardize the experiment wherever possible with proper acclimatization of the mice, preferably singly housed at the recommended room temperature of 28°C for 4 weeks in their respective cages for 12 h light/dark cycle with food and water *ad libitum* to compensate for the comfounding effects of social isolation. Obernier and Baldwin showed that a sufficient duration of acclimitization is required to recouperate from the physiological disturbances caused by transport (Obernier and Baldwin, 2006). In addition a duration of 4 weeks will allow the mice to adapt to the social isolation, the HLU cages, housing temperature.

It is important to monitor cortisol levels after suspension to ensure that the influence of stress is taken into account.

## Conclusion

The hindlimb unloading model developed in the 70s' to gain better understanding of the organismal response to microgravity, has then been adopted as a model to study other diseases such as disuse osteopenia.

The hindlimb unloading model has also contributed to identify potential drug candidates (e.g., Sclerostin antagonism using monoclonal antibodies) for the treatment of conditions such as osteopenia and osteoporosis. However, gaps still persist in our knowledge and understanding about the cellular and molecular pathways underpinning body response to microgravity. We caution on the importance of the experimental parameters that are adopted when running experiments based on murine hindlimb unloading. Overall, the murine hindlimb unloading still represents a key experimental model that willcertainly benefit the scientific community in the future to deepen our knowledge on pathophysiological processes that are of relevance for humans.

## Author Contributions

PG, MS, LP, LV, and DI wrote the first draft and carried out subsequent revisions. All authors contributed to the article and approved the submitted version.

## Funding

The authors acknowledge the financial contribution by the European Space Agency contract no. 4000128599/19/NL/PG, covering the MAP project BONUS - *In vitro* and *in vivo* pre-screening models and services for space and terrestrial interventions against bone and muscle fragility and CNES (Centre National d'Etudes Spatiales) contracts: no. 4800001065 OSTEOFLOW, no. 4800000966 ADIPOSTEO, and contract no. 4800000676 BION M1.

## Conflict of Interest

The authors declare that the research was conducted in the absence of any commercial or financial relationships that could be construed as a potential conflict of interest.

## Publisher's Note

All claims expressed in this article are solely those of the authors and do not necessarily represent those of their affiliated organizations, or those of the publisher, the editors and the reviewers. Any product that may be evaluated in this article, or claim that may be made by its manufacturer, is not guaranteed or endorsed by the publisher.
